# Development of Supervised Learning Predictive Models for Highly Non-linear Biological, Biomedical, and General Datasets

**DOI:** 10.3389/fmolb.2020.00013

**Published:** 2020-02-13

**Authors:** David Medina-Ortiz, Sebastián Contreras, Cristofer Quiroz, Álvaro Olivera-Nappa

**Affiliations:** ^1^Departamento de Ingeniería Química, Biotecnología y Materiales, Facultad de Ciencias Físicas y Matemáticas, Universidad de Chile, Santiago, Chile; ^2^Centre for Biotechnology and Bioengineering, Universidad de Chile, Santiago, Chile; ^3^Facultad de Ingeniería, Universidad Autónoma de Chile, Talca, Chile

**Keywords:** highly non-linear datasets, supervised learning algorithms, clustering, statistical techniques, recursive binary methods

## Abstract

In highly non-linear datasets, attributes or features do not allow readily finding visual patterns for identifying common underlying behaviors. Therefore, it is not possible to achieve classification or regression using linear or mildly non-linear hyperspace partition functions. Hence, supervised learning models based on the application of most existing algorithms are limited, and their performance metrics are low. Linear transformations of variables, such as principal components analysis, cannot avoid the problem, and even models based on artificial neural networks and deep learning are unable to improve the metrics. Sometimes, even when features allow classification or regression in reported cases, performance metrics of supervised learning algorithms remain unsatisfyingly low. This problem is recurrent in many areas of study as, per example, the clinical, biotechnological, and protein engineering areas, where many of the attributes are correlated in an unknown and very non-linear fashion or are categorical and difficult to relate to a target response variable. In such areas, being able to create predictive models would dramatically impact the quality of their outcomes, generating an immediate added value for both the scientific and general public. In this manuscript, we present RV-Clustering, a library of unsupervised learning algorithms, and a new methodology designed to find optimum partitions within highly non-linear datasets that allow deconvoluting variables and notoriously improving performance metrics in supervised learning classification or regression models. The partitions obtained are statistically cross-validated, ensuring correct representativity and no over-fitting. We have successfully tested RV-Clustering in several highly non-linear datasets with different origins. The approach herein proposed has generated classification and regression models with high-performance metrics, which further supports its ability to generate predictive models for highly non-linear datasets. Advantageously, the method does not require significant human input, which guarantees a higher usability in the biological, biomedical, and protein engineering community with no specific knowledge in the machine learning area.

## Introduction

In the so-called era of Data, Big Data seems to be a common term. As the name suggests, its determining characteristic is the amount of information, a quantity so large that it has required the development of new technologies and algorithms to obtain useful information from them (Katal et al., 2013; Sagiroglu and Sinanc, 2013; Gandomi and Haider, 2015). The above has attracted the interest of various actors, and among them, the field finds enthusiasts, detractors, and skeptics. In recent times, academic interest in Big Data revealed by the number of journals, conferences, and initiatives dedicated to the subject, has shown a consistently growing trend (Ekbia et al., 2015; Gandomi and Haider, 2015). From this increase, we can infer that, in addition to introducing new study directions and fields, Big Data has changed how research is carried out (Abbasi et al., 2016). The proliferation of information generators has created gigantic volumes and great diversity of data, and the evolution of the methods to analyze, store, transmit, and use them are radically reforming the scientific computing scenario (Hu et al., 2014; Asch et al., 2018; Oussous et al., 2018). Machine Learning (ML) techniques are an example of such methods (Al-Jarrah et al., 2015; Qiu et al., 2016; Zhou et al., 2017).

ML operates under the premise that it is possible to learn from the data and to generate predictions from the trends it may exhibit. ML, and any learning process in general, first involves a pattern discrimination stage, which is subsequently used for conjecturing predictions for new examples. Among the best-known ML methods, two separate groups can be drawn: supervised learning (Singh et al., 2016) and unsupervised learning (Ghahramani, 2003) methods. The first group of methods, usually associated with the classification and regression tasks, requires knowledge about a response variable, which is assumed to be related to and inferred from it. The second group of methods, generally related to clustering or pattern recognition tasks, does not require a previously known response variable since the output is clusters of behaviors that naturally emerge from the data (Witten et al., 2005). Examples of widely-used ML techniques are Artificial Neural Networks (ANN), Decision Trees (DT), Support Vector Machines (SVM), Naïve Bayes, *k*-nearest neighbors (KNN), and ensemble methods such as Boosting or Bagging, among others (Witten et al., 2005; Kourou et al., 2015). A general weakness of ML techniques, reported in different tenors, is an intrinsic part of their core: as they train from limited data, their results depend on their limited experience and, lacking a theoretical background, they frequently fail to cast predictions over exotic examples not present in the training set (Kourou et al., 2015; Michael et al., 2018). Some researchers commonly classify ML-trained models as “black boxes,” a term that results quite accurate for the ANN's applications (Olden and Jackson, 2002; Qiu and Jensen, 2004). However, models as DT, SVM, and KNN, for example, actually do rescue information about the decision-making workflow in their architecture, giving some insights about the reasons behind their results. In the area of biomedicine, where the applications are wide and very promising (Costa, 2014; Greene et al., 2014; Lee and Yoon, 2017), researchers call for a new era in the application of ML (Camacho et al., 2018), where the incorporation of information will be a key feature for success (Auffray et al., 2016; Michael et al., 2018). For instance, applications of ML may be found in studies related to cancer diagnosis and treatment (Kourou et al., 2015; Hinkson et al., 2017), diabetes research (Kavakiotis et al., 2017), decision support in critical care (Johnson et al., 2016), genomic medicine (Leung et al., 2015), among others.

Many times, the datasets do not have information about how their features interact to generate responses or clusters, which, added to the noise that datasets usually have, complicates its treatment. Researchers have pointed out this fact, emphasizing that it is difficult to bridge the gap between prediction and reality if the mechanistic background of the phenomenon to be predicted is not evident (Coveney et al., 2016). Depending on how complex the underlying relationships between the features are, classification or prediction models would be trained more or less smoothly. However, that complexity could also represent a prohibitive constraint, resulting in unacceptable performances of the trained models. Consequently, we may find natural that the success of ML techniques when training predictive models strongly rely on the data. In this work, we will call *linear datasets* those in which ML methods based on linearity assumptions generate models with *outstanding* performance measures. We will refer those datasets in which this does not happen as *non-linear datasets*. Some datasets result too complicated for linear models but may be suitable for applying mildly non-linear algorithms, such as non-linear Functional Data Analysis (FDA), Random Forest, AdaBoost, Gradient Tree Boosting, among others, or after performing a data pretreatment stage (Kourou et al., 2015). For this work, we will focus on those datasets in which, even after attempting to apply non-linear techniques, trained models do not reach acceptable performance. We will refer to these sets as *highly non-linear datasets*.

Previous works handling non-linear biological and biomedical datasets have used different Machine Learning-driven approaches to obtain predictors. Some of them use artificial neural networks (ANN) because of the high-performance metrics that these methods might achieve (Almeida, 2002; Rani, 2011; Shaikhina and Khovanova, 2017). Nevertheless, such performances can be altered by modifying the network hyperparameters (such as the number of layers or neuron units), often on the cost of overfitting the data. Other works have applied distance-based methods such as KNN (Ahmad et al., 2017), kernel-driven spatial transforms as SVM (Shi et al., 2013; Xiang et al., 2017), and variations of Partial Least Squares PLS (Sun et al., 2017), all after performing a specially tailored data pretreatment. This non-standard pretreatment results in the loss of generality of such approaches. Examples of the used data pretreatment techniques are classical Principal Components Analysis (PCA) and its variants, Factor Analysis (FA), and non-linear approaches as the t-distributed Stochastic Neighbor Embedding (t-SNE), Laplacian Eigenmaps and Locally Linear Embedding (LEM), and isometric mapping Isomaps (ISO), among others (Lee et al., 2008; Pandit et al., 2016; Rydzewski and Nowak, 2016; Doerr et al., 2017; Tribello and Gasparotto, 2019).

Since highly non-linear datasets are usually obtained while gathering scientific data, attempts have been performed using them to somehow develop predictive or interpretative models. However, these approaches lack generality as they have usually been developed for particular applications and used bare algorithms, which were combined with data pretreatment techniques, as described above, to increase performance metrics. Some of the examples we will use as study subjects in this manuscript relate to the fields of protein engineering, specifically stability assessment on point mutations (Capriotti et al., 2005; Masso and Vaisman, 2008; Getov et al., 2016) and protein localization in *E. coli* (Horton and Nakai, 1997; Zhang and Ling, 2001; Deshpande and Karypis, 2002; Ratanamahatana and Gunopulos, 2002), and clinical medicine, such as mammographic mass evolution (Elter et al., 2007) and thoracic surgery. Yet, the generation of a general methodology to treat these (highly) non-linear datasets in order to get predictive models is still an open problem, which we intend to tackle in the present manuscript.

Aiming to solve the model training underperformance issue over highly non-linear datasets, we present RV-Clustering, a library programmed in Python language, optimized for the development of predictive models for these datasets. In the following sections, the different modules implemented in the library and a new methodology to adequately obtain models in a highly non-linear dataset are described in detail. Following the workflow proposed by our methodology, the library implements different stages of data pretreatment and linearity assessment. In case the dataset is proven to be highly non-linear, the recursive binary partition, which is the central point of the algorithm, is carried out. The idea behind the method is the following: first, using unsupervised learning methods, a partition of the input dataset is generated. Afterward, different predictive models are locally trained in each subset, taking advantage of similarities among subset members to reach better performance metrics. After the local models are trained, they are validated and combined to form a meta-model. Before casting predictions on new cases, a global classification model is created to assign them to the subset where they belong, according to their features. The predictions result from applying the local meta-model on the new examples. We have successfully tested the proposed methodology in several highly non-linear datasets from a broad spectrum of origins, such as from the biomedical, biotechnology, and protein engineering areas. The versatility introduced by the proposed methodology highlights its potential benefits for users from all areas of knowledge, not only limited only to the fields mentioned above.

## Methods

Both the source code and the executable elements of RV-Clustering were implemented under the Python 2.7 programming language (Oliphant, 2007), mainly using the Scikit-learn (Pedregosa et al., 2011), Python Data Analysis (Pandas) (McKinney, 2011), and NumPy (Van Der Walt et al., 2011) libraries. The RV-Clustering library was designed under the Object-Oriented Programming paradigm (Wegner, 1990), aiming to provide the modularity required to perform actions separately in the proposed workflow. We tested the different functionalities of the library through the analysis of diverse datasets, mainly extracted from bibliographic reports of specific mutations in proteins and the effect they have on their properties and stability, and from open databases, such as BRENDA (Jeske et al., 2018), ProTherm (Bava et al., 2004), and the UCI Machine Learning repository (Dua and Graff, 2017).

## Overview of the RV-Clustering Methodology

RV-Clustering is a Python library, optimized for the creation and validation of predictive models for highly non-linear datasets. Its functionalities range from the typical data pretreatment techniques to the generation of predictive models for highly non-linear datasets. Our library stands out from others because of its ease of use, its modularity, the robustness of the implemented algorithms, and its open-source access. The details about the different commands and instructions for installing RV-Clustering in a local computer are available in the authors' Github repository (https://github.com/dMedinaO/nonlinearModels). Without being specific, RV-Clustering consists of different modules aiming to:

- Provide data pretreatment techniques.
- Assess the degree of non-linearity of the dataset.
- Create predictive models based on both supervised and unsupervised learning algorithms.
- Build and train meta-models.
- Generate partitions of the dataset, where models reach high performances more efficiently while being trained.
- Evaluate performance metrics of the implemented models.

To highlight the motivation behind the proposed library and methodology, we will explain its different modules as they appear in the proposed workflow. Briefly, RV-Clustering modules for the treatment of highly non-linear datasets are based on a recursive binary partition of the initial dataset and subsequent training of the predictive models for assigning new examples to the constitutive subsets. Afterward, RV-Clustering generates different predictive models within the resulting partition, generating a battery of local models that predicts examples inside the subset. When the user wants to evaluate a new example, RV-Clustering assigns it to one of the subsets within the partition, and then the local models cast the predictions to form the output. RV-Clustering also reports the performance metrics and statistical analyses of the resulting classification model, the within-the-partition local models, and the general meta-model.

**Algorithm 1:**
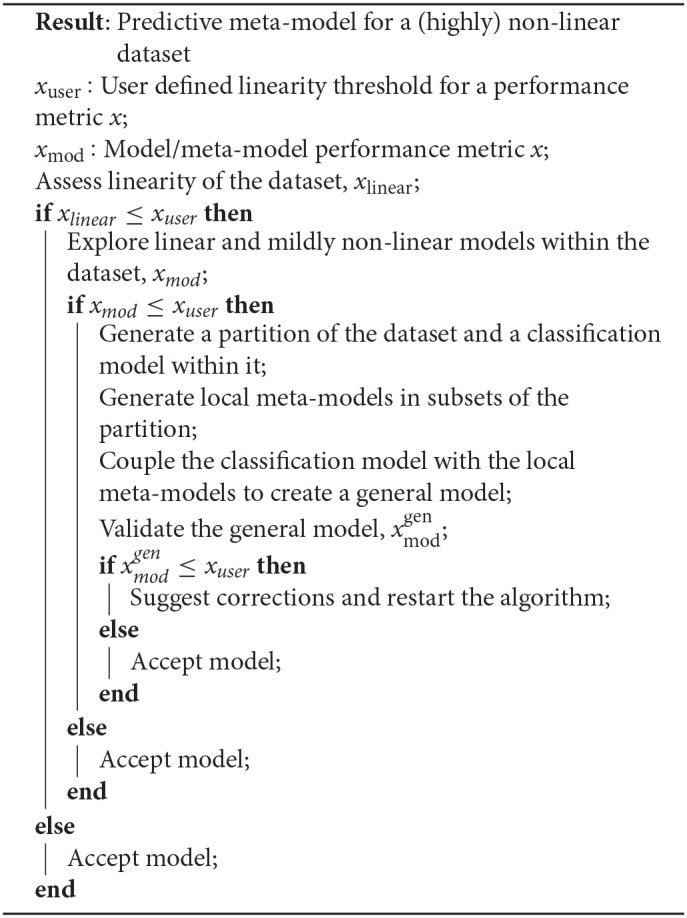
RV-Clustering methodology

## Results

###  RV-Clustering Modules Through the Proposed Methodology

**Figure 1:**
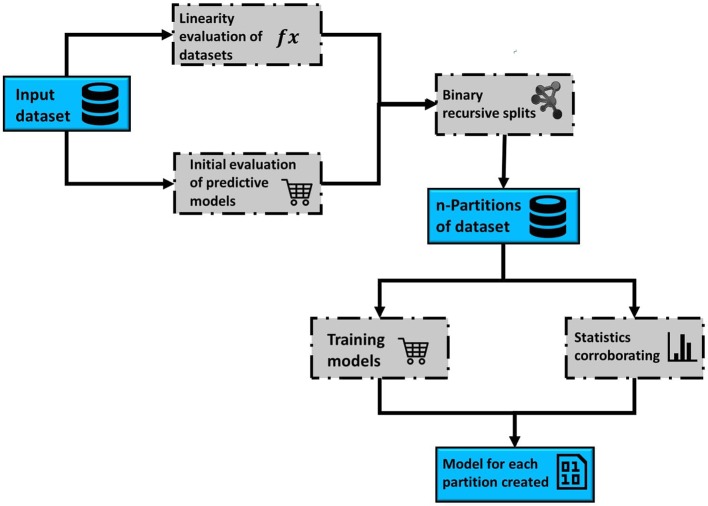
Representative scheme of the workflow associated with the methodology proposed to develop predictive models for highly non-linear datasets, based on the use of the RV-Clustering library.

This section comprises the description of the different modules implemented in the RV-Clustering command library and the proposed methodology. Figure 1 represents the workflow of our method. As an input, RV-Clustering receives the dataset and configuration parameters for the evaluation of different criteria such as the minimum percentage of elements in each group, the kind of model to be trained, and the minimum ratio accepted for the detection of class imbalance, in the case of classification models. At this stage, the user also must declare thresholds to evaluate whether the dataset is considered as linear or non-linear, and minimum expected performance metrics in the exploratory stage of predictive models.

#### Data Preprocessing

RV-Clustering incorporates a dataset preprocessing stage that allows encoding categorical variables using One Hot Encoder and assessing the class imbalance, if applicable. Finally, RV-Clustering standardizes the dataset and divides it into two groups: a training subset (80% of the original dataset) and a validation subset (the remaining 20%).

#### Evaluation of Dataset Linearity

In the first instance, RV-Clustering evaluates whether the dataset is non-linear according to our definition. To do this, the user must indicate if the desired model is for or classification. If the models to be trained are regression models, the tool applies a linear regression on the dataset based on ordinal least squares and obtains the coefficient of determination value of the result (*R*^2^). Otherwise, it applies a variation of the Ho-Kashyap algorithm (Serpico and Moser, 2006), in which different linear classification methods, based on Support Vector Machines (SVM) and its variants, are implemented. Finally, we compare the accuracy of the obtained models with the minimum acceptance threshold defined by the user. Thus, any dataset that does not meet this criterion is classified as non-linear and is a candidate to undergo the process of recursive binary partitions.

#### Initial Exploration of Predictive Models

RV-Clustering allows the user to perform an exploratory stage for testing the performance metrics of predictive models based on supervised learning algorithms. This evaluation receives as input: (i) the dataset, (ii) the performance measure of interest, (iii) the minimum performance threshold, (iv) the type of response (categorical or numerical), and (v) the response column identifier.

To perform the exploration, the model training module of our tool applies different supervised learning algorithms to the dataset, depending on the type of response. After training the models, we obtain distributions of performance metrics, selecting the model with the highest performance according to the user-input metric. If the performance is higher than the threshold declared by the user, the tool reports as output the respective model and all its performance metrics. Otherwise, a message informing that no model meets the desired requirements will appear. If that were the case, there are two different actions to take that may help to reverse the result: (i) reducing the dimensionality of the dataset by selecting the most informative attributes or, on the contrary, (ii) adding further information to the dataset. The first requires knowledge about the available techniques for dimensionality reduction, while the addition of information may not be favorable if it is not informative enough and only serves to increase the noise in the dataset. Finally, if none of the options works, it is recommended to submit the dataset to the recursive binary partition stage proposed in this work.

It is essential to mention that this stage is complementary to the evaluation of the linearity of the datasets since the contemplated algorithms are not linear regressions or hyperplane generation-based. Alternatively, we instead employ probability distributions (Naïve Bayes and derivatives), evaluation of characteristics (Decision Trees), or boosting methods (Random Forest, Adaboost, Bagging, Gradient Tree Boosting) for model training.

#### Recursive Binary Partitions

The main objective of the recursive binary partition process is the generation of subsets from the initial dataset, wherein we could increase the performance metrics previously obtained in the exploratory stage of supervised learning models. A binary search trees-inspired algorithm (Bentley, 1975), where the search is optimized in the tree path, generate the partitions. In each iteration, the initial dataset is subjected to an exploration of different unsupervised learning clustering methods, such as the Birch, *k*-Means, and Agglomerative algorithms, conditioned to the generation of two elements. In the cases of *k*-Means and Birch, our algorithm automatically tests different distance metrics, while for Agglomerative Clustering, the affinity parameter and linkage methods are automatically varied. Each proposed partition is evaluated using the silhouette coefficient and the Calinski-Harabasz index. Subsequently, we evaluate the number of subset elements of those partitions that have the highest clustering performance indexes. The number of elements in each subset should be equal or higher than the minimum threshold previously selected by the user. Class imbalance generated by the partition is assessed according to a user-determined threshold for classification models. Finally, if the partition in a given iteration meets all the mentioned criteria, it is accepted, and the recursive division continues for each tree branch. At the end of the execution, we will have *n* subsets, which will be statistically studied to evaluate if each generated partition is significantly different from the others, if each element effectively belongs to its corresponding subset, and if all the features are informative for all subsets, in order to avoid any redundancy that could affect the model training stage.

#### Creation of Models to Classify New Examples in the Generated Partition

In order to classify examples within the generated partition, different classification models are created, using supervised learning algorithms. For this, the training dataset, which is already a subset of the input dataset, is divided into two sets for training (80%) and validating (20%) the classification models. The first subset undergoes a model exploratory stage training with *k*-cross-validation, with *k*-values varying depending on the size of the set. We obtain the accuracy, recall, precision, and F1 scores for each model, and also their statistical distributions. From these four distributions of performance metrics, the models with the maximum values in these distributions are selected, forming a set of at most four independent models (one per each performance metric). These four models are used to generate a weighted meta-model with a classification criterion obtained by the votation of the individual models, assigning each element to the subset pointed by the majority of the individual models. Finally, we compare the classifications generated by the meta-model with the actual values of the validation set to obtain the overall performance metrics.

#### Model Training

Each subset *A*_*i*_ within the partition generated in the binary recursive division undergoes a predictive model exploration stage, and the best *j* models are selected and combined to form a local meta-model. The selection criterion is associated with the maximum value of each metric of interest selected by the user, which may be accuracy, recall, precision, or F1 for classification models, or *R*^2^, Pearson, Kendall τ, or Spearman rank coefficients for regression models, hence *j* ≤ 4. RV-Clustering estimates an overall performance for the models over the entire dataset, weighting the individual metrics in the generated partition. Let *x*_*i*_ be a metric of the models' performance over *A*_*i*_. The corresponding *i*-weighted performance is given by

(1) $$\begin{array}{l} {\hat{x}}_{i}=x_{i}\cdot \frac{\left|A_{i}\right|}{\left|\overset{n}{\underset{i=1}{⋃}}A_{i}\right|}, \end{array}$$

and the final measure is obtained from the summation of the ${\hat{x}}_{i}$, which corresponds to the probabilistic expected value of *x, E*(*x*) assigning a probability $\mathbb{P}\left(A_{i}\right)=\frac{\left|A_{i}\right|}{\left|{⋃}_{i=1}^{n}A_{i}\right|}$ to the subset *A*_*i*_,

(2) $$\begin{array}{l} \hat{x}=\overset{n}{\underset{i=1}{\sum }}{\hat{x}}_{i}=\overset{n}{\underset{i=1}{\sum }}x_{i}\text{P}\left(A_{i}\right)=\text{E}\left(x\right). \end{array}$$

We compare the obtained weighted measure with the performance values obtained in the initial stage, reporting them both. Finally, the tool uses the validation set to obtain the real metrics *x*genmod of the general model created, and report the results associated with the classification or prediction of new examples. To do this, RV-Clustering uses the classification model to assign each example to the subset in the partition where it should belong, and then, using the local meta-model corresponding to that subset, obtain the predicted value. We compare this value with the real value and generate the performance metrics corresponding to the type of model.

An index for assessing over-fitting local meta-models within the partitions *IOF* is presented in Equation (3), defined as the difference between the expected (via Equation 2) and the real performance metric.

(3) $$IOF=\frac{\hat{x}-x_{\mathrm{mod}}^{\text{gen}}}{x_{\mathrm{mod}}^{\text{gen}}},\;IOF_{i}=\frac{x_{i}-x_{\mathrm{mod}}^{\text{gen}}}{x_{\mathrm{mod}}^{\text{gen}}}$$

Similarly to Equation (1), it is possible to obtain a local *IOF* for subset *i*, *IOF*_*i*_. If the *IOF* or any of the local *IOF*_*i*_ values are >5% or another user-customizable value, the recursive binary partition algorithm should be repeated, conditioned to producing subsets with more elements. Negative values of *IOF* do not have any implications, as they only show that the performance of the global model is greater than the expected value, accounting for a synergy between individual meta-models.

#### Predicting New Examples

The proposed method creates a partition splitting the input dataset into *n* subsets. Hence, as we work independently in each subset, we obtain *n* independent meta-models. In order to classify new examples within the subsets of the obtained partition, we train a classification model, which assigns every new example to the subset where it should belong. For this, RV-Clustering classifies the new example into a particular subset in the partition, applying the predictions of local meta-models. We can directly calculate the improvement of the original result *I* from the linearity assessment index and the final performance metric,

(4) $$I=\frac{x_{\mathrm{mod}}^{\text{gen}}-x_{\text{linear}}}{x_{\text{linear}}}$$

## Cases of Study

The proposed methodology and library modules were tested with different highly non-linear datasets according to our previous definition, related to clinical diagnosis, biotechnology, and protein engineering. Each one of the proposed scenarios is presented below in three different case studies.

### Case Study I: Use of RV-Clustering in Clinical Datasets

The prediction of the clinical risk associated with mutations in proteins, the probability of having a disease, or the need to carry out an invasive or dangerous exam, among others, are activities of high interest in the biomedical area. Taking this into consideration, the different points of the methodology proposed in this article were applied to three highly non-linear datasets, which represent Mammographic Mass, Heart-Disease, and Thoracic Surgery. The datasets were extracted from the UCI-Machine Learning (Dua and Graff, 2017) repository and, in all cases, the required models are of the classification type, since their response is categorical.

**Figure 2:**
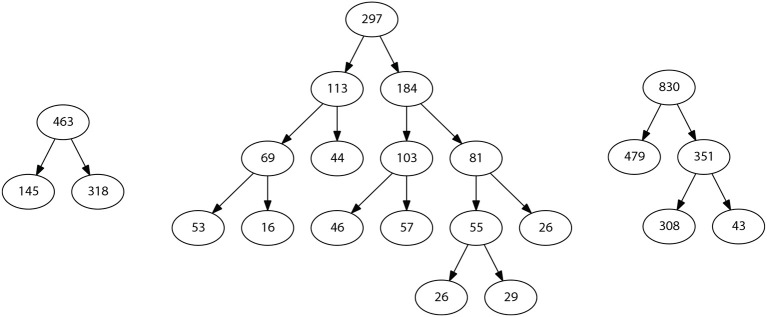
Representative schemes of the partitions and the flows of divisions generated for the example datasets associated with case study I: Thoracic Surgery dataset **(left)**, Heart Disease Cleveland dataset **(center)**, and Mamographic Mass dataset **(right)**. The number of final partitions, their cardinality and the performance measures achieved by the models trained in each case are also presented.

When performing the linearity assessment, all the datasets turned out to be highly non-linear, considering a minimum threshold of 0.8 for the linearity metrics. This stringent criterion was selected to impose a high quality of the classification since false positive and false negative errors should be as low as possible for a clinical test. The performances obtained in the model exploration stage using mildly non-linear methods did not reach the minimum threshold values, so RV-Clustering proceeded to apply the binary partition methods proposed in this work. Figure 2 shows the partition generated for each dataset. In each case, the cardinality of the generated subsets varies as the depth of the resulting binary tree increases. The performance metrics obtained for Mammographic Mass and Thoracic Surgery models applying the proposed methodology is considerably greater than those obtained in the exploratory stage since accuracy is improved from 54 to 87% in the first case, and from 71 to 83% in the second case. In the Heart-Disease Cleveland dataset, no considerable improvement was achieved. We consider this to be due to the large number of classes presented by this dataset. Given this result, as RV-Clustering ensures class balance in each subset within the partition, the recursive binary partition method should not be used with datasets whose response categories are >5, especially when the number of examples is limited, because it may lead to detriments on the performance metrics initially achieved. This limitation arises from the lack of information in the dataset itself, as the generation of regressions or predictions of high-dimensional responses based on few data examples remains an open problem.

### Case Study II: Use of RV-Clustering in Biotechnological Datasets

**Figure 3:**
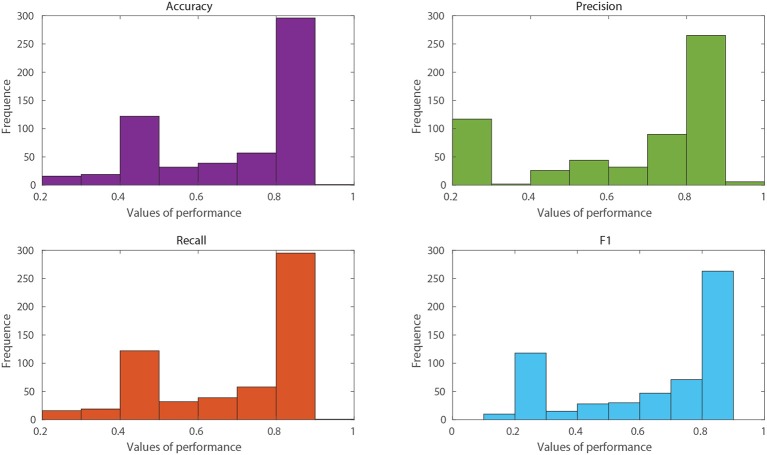
Histograms of performance metrics obtained in the exploration stage by the RV-Clustering library for the protein location in an *E. coli* dataset. The highest values were obtained by methods based on Bagging or Boosting algorithms, accounting for the non-linearity of the dataset.

Another approach of a broad interest in the use of data mining and ML techniques is the development of predictive models for the optimisation of experimental plans in biotechnological applications. Through the generated predictive models, it is possible to reduce the use of economic and human resources and the duration of the experimental projects dramatically. As an example, a dataset with information on the classification of protein localisation sites in *E. coli*, extracted from the UCI Machine Learning repository (Dua and Graff, 2017), will be used. This dataset was subjected to the linearity assessment, contemplating a minimum acceptance threshold of 0.7 in linearity metrics. As the highest accuracy achieved was 56%, RV-Clustering classified this dataset as non-linear. However, when applying the model exploration module, satisfactory results were obtained. The distributions presented in Figure 3 show a set of models that have performance measures greater than those of the threshold imposed. Hence, it is not necessary to proceed to the binary recursive partition stage. The best models trained in the exploration stage are selected to create a weighted meta-model, whose accuracy and precision reached 88.1 %.

In particular, given the properties of the input dataset, it was possible to obtain a meta-model with performance metrics above those imposed as an experimental requirement, only by applying the exploratory module. This fact highlights the efficiency of RV-Clustering, always aiming to satisfy the user requirements to obtain as-good-as-required models as fast as possible and without incurring in greater trade-offs in quality-time. Using the modules implemented in RV-Clustering, it was possible to improve the initial accuracy of 56% to a value of 88.1%, confirming that the proposed workflow is appropriated. It is crucial to know which algorithms are the most suitable for a given application, and it is a great advantage of RV-Clustering to test them in such a way that all the possibilities are evaluated, without requiring any specific knowledge on algorithms for getting high-quality results.

### Case Study III: Use of RV-Clustering for the Evaluation of Protein Stability Given Point Mutations

**Figure 4:**
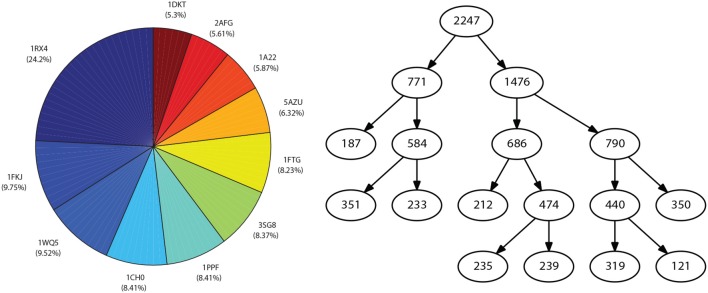
Representation of the dataset associated with case study III: Distribution of mutations for the considered proteins **(left)**, and Resulting partition after applying the methodology proposed in this work **(right)**.

The evaluation of the effect that point mutations have in protein stability is one of the most visited topics in protein engineering. Different approaches have been proposed, considering methods based on electrostatic potentials, statistics, ML techniques, among others. The methods mentioned above allow a mutation to be classified as stable or non-stable or to generate stability predictions based on the difference in free energy (ΔΔ*G*) caused by the replacement of the residue. Applying the approach proposed by Capriotti et al. (2005) for describing mutations and considering three independent descriptors, thermodynamic, structural and residue-environmental, a dataset comprising 11 proteins and 2,247 mutations associated was generated (see Figure 4, left). In the created dataset, the response column represents the ΔΔ*G* values, associated with the difference between mutated residue and wild residue. These values were obtained from the ProTherm (Bava et al., 2004) database.

**Figure 5:**
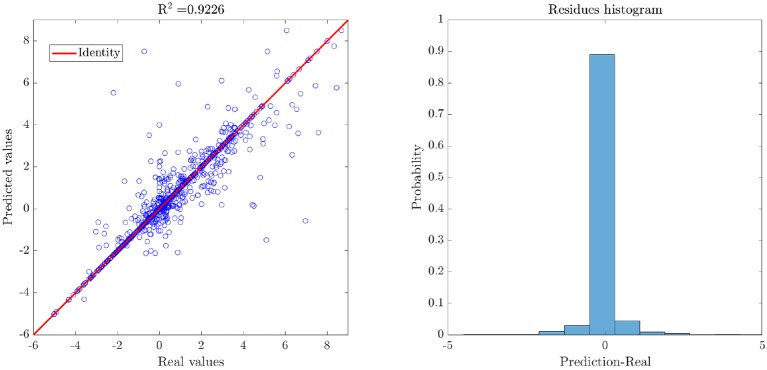
Results of the generated weighted meta-model, where the predicted values are obtained from the average of the predictions of the individual methods. As the error seems to have a random distribution around zero, ΔΔ*G* values predicted by the meta-model do not present considerable biases.

The application of the linearity assessment module classified the dataset as non-linear, since the performance metrics obtained by applying linear methods did not exceed the threshold of 0.6 for predictive models. Furthermore, as it was not possible to achieve significantly higher performance measures in the model exploration stage, the dataset was classified as highly non-linear. By applying the proposed methodology for binary recursive partition, nine subsets were obtained (see Figure 4, right), and different meta-models were developed locally. Intra-partition over-adjustment was avoided by applying a *k*-cross-validation, with *k* = 10. Subsequently, a meta-model for the classification of new examples to the different partitions was generated. Finally, the general metrics of the model were obtained for the validation set (see Figure 5, left). By comparing the resulting performance metrics and the initial values obtained in the exploration stage of predictive models, an average improvement of 40% was achieved in each measure of interest. For example, the initial Pearson's coefficient of 0.58 was improved to 0.92 after applying the methodology here presented. A scatter plot of the real and predicted values for the effect of point mutations shows that the error distribution has a random and bounded behavior (see Figure 5, right), which corroborates the quality of the obtained results.

## Discussion

###  Improvements on Performance Metrics

The different datasets tested in the cases studies serve to illustrate the great capacities of the proposed method since it not only improves the performance measures, but it does so efficiently from a computational point of view, generating as-good-as-required models in the shortest time possible. This result is achieved thanks to the RV-Clustering library modularity and the structure of the presented methodology, which considers advancing to the next complexity level only when models generated so far do not meet user requirements.

Another advantage of this new approach is the transparency of the results. Model performance metrics, by themselves, may not be sufficiently informative and mislead to wrong conclusions about the quality of the predictive outcome; they should always be analyzed in context. In our work, the different metrics associated with different elements (models, meta-models, global model) are analyzed together and combined using the proposed indexes. This combination of metrics is used both for improvement evaluation between the initial linear assessment stage and the final performance and for the evaluation of over-fitting in local meta-models within the partition. Table 1 presents the results of the considered cases of study, all of which show a significant improvement in their metrics. No over-fitting of the local meta-models was observed in the different subgroups of the partition since all *IOF* values were negative. The previous discussion also accounts for synergistic effects between the classification model and the different meta-models within the partition, since overall performance metrics are higher than weighted individual ones. All of the above translates into an average percentage increase of 47.3% in the performance metrics of the predictive models for the highly non-linear biological datasets considered, as presented in Table 1. As the performance metrics increase as the methodology proceeds, the best model will always be the latest delivered (except in cases where *IOF* > 0). To stop at early stages by imposing lower values of *x*user is a decision based on a time-quality trade-off, as our methodology was thought for delivering as-good-as-required models.

**Table 1:** Evolution of performance (accuracy) of the models generated in different progressive steps of the proposed methodology.

**Dataset**	***x*_linear_**	**$\hat{x}$**	**$x_{\mathrm{mod}}^{\text{gen}}$**	**Improvement after applying RV-Clustering's methodology (%)**	***IOF*(%)**
Mammografic mass	0.54	0.85	0.87	61.1	−2.3
Thoracic surgery	0.71	0.78	0.87	22.5	−10.3
Protein location in *E. coli*	0.56	–	0.88	57.1	–
Protein stability^*^	0.58	0.82	0.92	48.3	−4.7

*The performance of the final RV-Clustering generated model is represented by $x_{mod}^{gen}$, while x_linear_ and $\hat{x}$ are the results of intermediate steps of the method (linear assessment step and model exploration step, respectively)*.

* *Pearson's coefficient*.

Table 2 presents the overall improvement in the performance metrics after applying our methodology, compared to the values reported in the original works. As our methodology incorporates most of the best state-of-the-art available algorithms and progressively applies them, the worst scenario would always be better than the original one.

**Table 2:** Comparison of reported performance metrics for the studied experimental datasets.

**Dataset**	**Reported by**	**Reported performance**	**RV-Clustering performance**
Protein stability (point mutations)	Capriotti et al., 2005	0.71	0.92
	Deshpande and Karypis, 2002	0.73	
Classification of protein location in *E. coli*	Zhang and Ling, 2001	0.84	0.88
	Horton and Nakai, 1997	0.68	
	Ratanamahatana and Gunopulos, 2002	0.84	
Mammographic mass	Elter et al., 2007	0.87	0.87
Thoracic surgery	None	None	0.87

###  Testing on Artificial Datasets

In order to test the proposed methodology and the robustness of our library, we generated different artificial datasets with tailored properties, aiming to evaluate its response against (a) noise intensity, (b) presence of outliers, (c) degree of non-linearity of the input dataset, and (d) maximum dimension of the input dataset, with further recommendations based on the fitting procedure.

Given that our methodology is very intuitive to understand when applied to regression models (as discussed in section 3), all models trained in this section were of the regression type. We explain each of the cases in the subsections below.

#### Noise Intensity

To show the influence of noise intensity or experimental errors, we tested our methodology with two different datasets: an artificial dataset containing a linear ground truth function, and the dataset of Case Study III. We introduced an additive proportional error to the response variable, characterized by a variable amplitude α. Adding this error to the experimental values *y*_exp_ resulted in the following expression for $y_{i}^{\text{noise}}$:

(5) $$y_{i}^{\text{noise}}=y_{\exp}(x_{i})(1+\alpha (2U-1))$$

where U is a random variable with uniform values in [0, 1]. Equation (5) was selected because of its statistical properties, given that the expected value of the noisy random variable is its corresponding experimental value:

E(yinoise)=yexp(xi)(1+α(2E(U)−1))               =yexp(xi)(1+α(212−1))               =yexp(xi).

**Figure 6:**
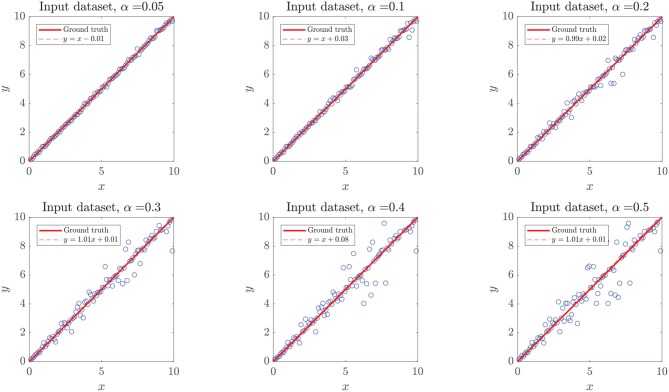
Simulated dataset with added white noise α. The plots represent simulated (y) vs. ground truth (x) data points (circles), the identity line (continuous line), and the crude statistical regression of the resulting dataset (discontinuous line). Added noise followed a Gaussian distribution around the expected value *y* = *x*, not affecting the expected value of the distribution, which translates to regression lines very similar to the identity.

Aiming to test how heavily the increasing noise impacts the performance metrics, we considered two scenarios: (a) adding α-noise to the experimental ΔΔ*G* data (Case of Study III), classified as highly non-linear, with an unknown ground truth function, and (b) adding α-noise to numerical experiments with known ground truth *y* = *x*, which included a white Gaussian noise with σ = 5%, in order to resemble real-world measurements. For both scenarios, we considered α ∈ [5, 10, 20, 30, 40, 50%], as shown in Figure 6.

**Figure 7:**
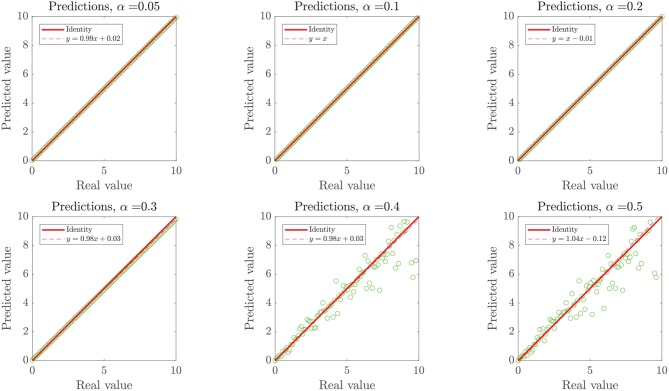
Model-prediction of the simulated linear dataset with α− induced noise in 100 data points. The plots represent predicted (y) vs. real (ground truth without noise, x) data points (circles), the identity line (continuous line), and the crude linear statistical regression of the scatter (discontinuous line). Since training datasets for models included noise, we expect particular discordance between the dispersion of high α scenarios and the predictive outcome of noise-fitting models trained therein, when compared to the original noise-free dataset.

**Figure 8:**
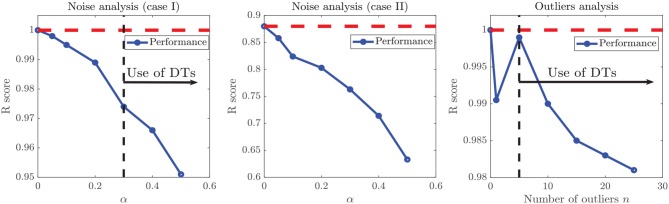
Evolution of model performance metrics against noise. **(Left)** Model performance on a linear ground truth function with white noise. **(Center)** Model performance on experimental data (Case Study III) with white noise. **(Right)** Model performance on a linear ground truth function with different number of outliers. In artificial datasets with linear ground truth functions (left and right images) *x*_user_ was set equal to 0.95 to force the algorithm to continue further in the proposed methodology. When the linear model performance fell under the selected threshold, the algorithm swap to DT models, which rose the performance metrics again, generating a break in the sloping trends.

In the first case, as the ground truth function is linear, we set *x*_user_ = 0.95 to force our algorithm to move forward into the second step of our methodology. However, even when the noise intensity was α = 20%, models generated in the first step of our methodology (linear assessment stage) still reached performance metrics over the threshold *x*_linear_ > *x*_user_. When the noise intensity was higher, linear models did not meet the required performance, but those generated by DTs and RF did, preventing the algorithm from entering into the binary splitting stage. Despite the generated models reaching high-performance measures at every α−noise scenario (see the left plot in **Figure 8**), a bifurcation in the quality of the predictive outcome appears when the nature of the training algorithms shifts from linear regressions to DTs. As shown in Figure 7, the scatter plot of predictions and ground truth (original data without noise) present high dispersion when α ≥ 40%, even though models reached high performance metrics, which accounts for models fitting the noisy data rather than the original trend. The above highlights the need for a preliminary analysis of the data, as moderate to high noise can mislead the results and affect the quality of the produced models. However, a 40% or higher noise level is large by any measure, and would not be usually considered as simple noise but rather as a composition of signals. In this sense, the fitting given by our algorithms in the presence of high “noise” points into the right direction by identifying the data points as coming from a model different from the linear ground truth function. The outstanding predictions of the models generated at low α can be explained by the linear nature of the ground truth.

When the considered dataset was classified as highly non-linear, added noise had a stronger impact on performance metrics, as shown in the center graph of Figure 8. In this case, the range of the *y*_−axis_ is much wider than in other cases. Noise levels over α = 20% have a more significant impact over performance metrics, since the slope of the α vs. performance curve is always decreasing. Such impact can be assessed from the decrease in the improvement after applying the RV-Clustering methodology (see the sixth and seventh row of Table 3). Given that noise levels under α = 20% do not have a severe impact on the performance metrics of the generated models, we show our methodology to be robust against low to moderate white noise.

**Table 3:** Evolution of performance (accuracy) of the models generated in different progressive steps of the proposed methodology, applied to noisy variations of the dataset used in Case Study 3.

**Induced noise α[%]**	***x*_linear_**	**$\hat{x}$**	**$x_{\mathrm{mod}}^{\text{gen}}$**	**Improvement after applying RV-Clustering's methodology (%)**	***IOF(%)***
0	0.58	0.82	0.92	58.62	−10.87
5	0.57	0.79	0.86	51.86	−7.58
10	0.55	0.78	0.82	48.74	−5.22
20	0.55	0.77	0.80	47.34	−3.86
30	0.54	0.75	0.76	41.04	−1.57
40	0.53	0.69	0.71	33.96	−3.64
50	0.52	0.59	0.63	22.44	−6.32

#### Presence of Outliers

**Figure 9:**
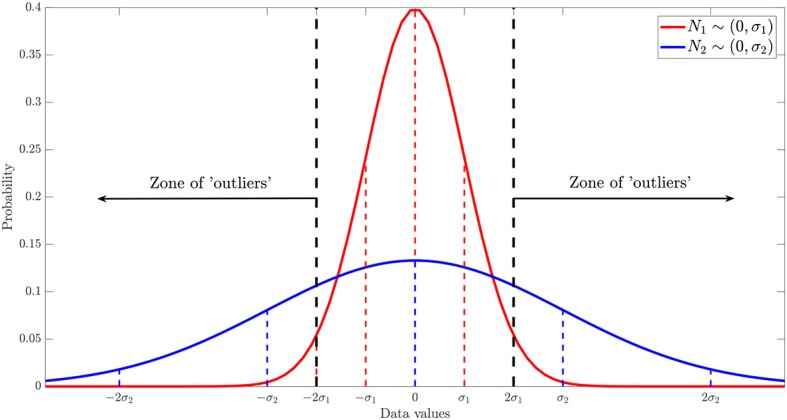
Scheme of the non-arbitrary and statistical methodology proposed to generate outliers, given a known dataset with random error.

To evaluate the robustness of our methodology and command library against the presence of outliers in the dataset, we performed the following numerical experiment. Starting with data with a known ground truth function, *y*(*x*) = *x*, we added a white Gaussian noise $N_{1}\sim N\left(0,\sigma_{1}=0.25\right)$. Hence, our “experimental” dataset was the collection of random variables $y_{i}^{\text{noise}}∼N(x_{i},\sigma )$. To simulate the existence of *n* outliers, we superposed a flat Gaussian distribution $N_{2}\sim N\left(0,\sigma_{2}\gg \sigma_{1}\right)$, as depicted in Figure 9, and applied the method described in the Algorithm 2.

**Algorithm 2:**
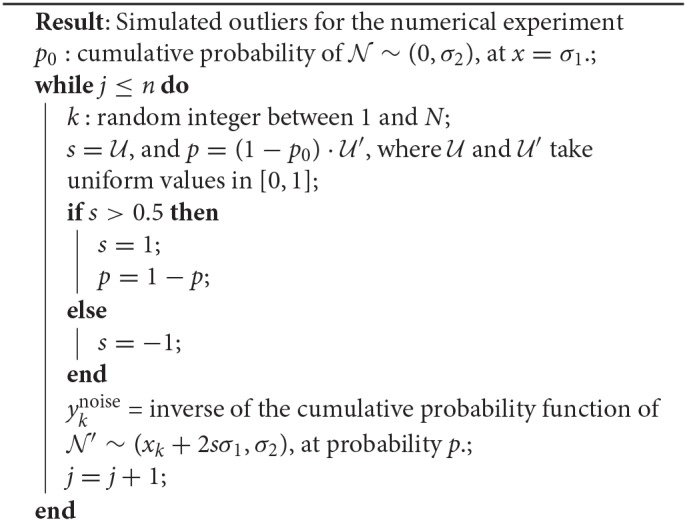
Numerical experiment with simulated outliers.

**Figure 10:**
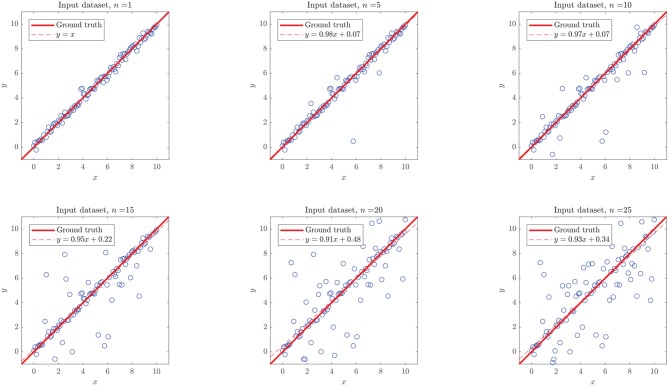
Simulated linear dataset with *n* outliers in 100 data points. The added outliers modify the nature of the distribution, as they make the regression considerably drift from the identity. In such sense, those outliers might not be “pure” in a strict statistical definition (as they depend on the distribution), they drift considerably from the expected values of the original distribution.

We simulated different datasets of *N* = 100 examples, and turned *n* of them into “outliers,” with *n* = {1, 5, 10, 15, 20, 25}, as shown in Figure 10. Noticeably, the added outliers modify the nature of the original Gaussian distribution, which is demonstrated by the drift between the identity and the purely statistical regression of the data points as more outliers are added to the dataset. In such sense, those outliers drift considerably from the expected values of the original distribution. Nevertheless, the presence of less than ~10% outliers does not affect the performance of the final model. Even when outliers are not symmetric (see examples with 5, 10, and 15 outliers in Figure 10).

As shown in the right plot of Figure 8, the presence of outliers negatively affects the linearity of the dataset as perceived by the methodology, since linear models do not meet the required performance and the RV-clustering workflow would move forward to DTs and non-linear algorithms. Nevertheless, and once again because of the linearity of the ground truth function, DTs would produce models with outstanding performance, producing a clear break in the sloping trend of the *n* vs. performance curve of Figure 8 and preventing the algorithm to proceed to the recursive binary splitting stage. When a high number of outliers are expected within the dataset, we recommend to directly proceed to probability-based methods by setting a high *x*_user_ threshold.

#### Degree of Non-linearity of the Input Dataset

To evaluate the robustness of our methodology and command library against the degree of non-linearity of the ground truth function, we simulated different points from the 2-D Rosenbrock function (Rosenbrock, 1960), with parameters *a* = 5 and *b* = 2, over the [0, 3]^2^ rectangle. Data for the numerical experiment were randomly extracted from the [0, 3]^2^ rectangle, and a proportional white Gaussian noise was added to resemble experimental conditions. When setting a threshold *x*_user_ = 0.9 the dataset would be classified as non-linear, and the methodology would proceed to explore non-linear algorithms for training models. Among the algorithms that produced models with outstanding performance metrics, we found DTs (0.998), Bagging (0.995), Random Forest (0.995), KNN (0.98), and Adaboost (0.95), with an over-fitting assessment of *k*-cross-validation, *k* = 10. As expected, given the non-linear nature of the ground truth function of the dataset, the best performing algorithms mentioned above are based on feature analysis, bagging, or boosting. In particular, we expected KNN to be within the outstanding algorithms, given its distance-based generation of predictions, although it occupies only the fourth place among the best predictors.

**Figure 11:**
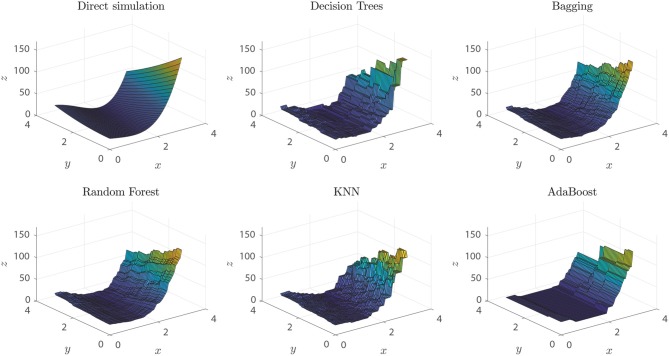
Rosenbrock function predictions of each of the different models generated. Direct simulation represents the real ground truth Rosenbrock function.

Visually, we can corroborate that the best models were those based in DTs, Bagging and Random Forest algorithms (see Figure 11). All these models are able to predict extreme values of the function, the local maximum at (0, 3), the valley of minimum values at (*x, x*^2^) and the extreme values around (3, 0). Random Forest and Bagging model predictions are smoother than other models and are good to predict function values in sectors with higher slopes and variability. Smoothness in this frame can be interpreted as a measure of the model insensitivity to noise, which points to Random Forest models as the best ones in this respect.

#### Maximum Problem Size, Properties of the Input Dataset, and Further Recommendations

We tested different cases where the dimensions of the input dataset were progressively increasing, aiming to determine a size threshold for the datasets RV-Clustering may process in a reasonable time. Our exploration found special cases where the input datasets may produce errors. The maximum dataset size that can be processed is less than 10, 000 × 1, 000, i.e., 10,000 examples with 1,000 features. In the current implementation of RV-Clustering, when submitting a dataset with such dimensions, more than 16 GB of RAM are used, which results in process abortion. To prevent the situation mentioned above, we suggest applying a dimension reduction technique prior to using our methodology, and taking the resulting dataset with fewer features as the input dataset for RV-Clustering. As maximum execution time, a dataset with 10,000 examples and 500 features would take 6 days and 2 h to be processed by a seventh generation Intel Core i5 processor.

As further recommendations and good practices for using the RV-Clustering tool, we suggest:

- Standardizing numerical datasets with float size less or equal than 64.
- Keeping in mind that categorical datasets where the number of features is >20% the number of examples would be coded using One Hot Encoder, hence consuming more resources and taking much more time to be processed.
- Carefully “refining” user datasets before submitting a job to RV-Clustering. For example, numerical datasets with alphanumerical entrances would stop the process, and a warning message would pop-out.
- Especially in the case of regression models (which are not “protected” with a class balance assessment), procuring that data is well-distributed and there are no information gaps in the predictor variables. Not taking care of this situation may lead to poor fitting of the un-populated zones or filling-in with erroneous predictions if unattended, respectively. The first point can be corrected by pre-processing the data to collapse the populated zones into fewer data points to balance their weights, or selecting a different performance metric as the control variable. For the second point, unfortunately, it is not possible to find an always-working solution: as we do not know *a priori* the real values of the data in the unpopulated zone, the errors in the predictions are unbounded. We can avoid this fact being a problem for the algorithm by splitting the dataset in parts, and processing each subset separately, or forcing the algorithm to proceed to the binary splitting stage. However, this solution will not give any model prediction for the unpopulated gap zone in the original data.

## Conclusions

We presented a new methodology for the design and implementation of classification or regression models for highly non-linear datasets, together with the RV-Clustering library, which corresponds to a set of modules implemented in Python that allow the manipulation of these datasets and the training of predictive models through supervised learning algorithms. This new methodology is based on a binary recursive division of the dataset, in order to generate subsets in which it would be possible to train predictive models with higher final performances, taking advantage of similarities between members. In each subset of the generated partition, models are trained, and the best ones are combined to form a meta-model. Separately, a model to classify new examples within the subsets in the partition is created. Finally, we generate a global model that assigns new examples to a particular subset using the classification mentioned above model, and predicts their value using the local meta-model for each case.

We successfully tested this new method in different non-linear datasets from different origins in the clinical, biomedical, biotechnological, and protein engineering fields. On those datasets, predictive meta-models were created, and high performance metrics were achieved, far above those obtained with other methods. The use of numerical experiments helped us to test the boundaries of our methodology, controlling the predictive outcome and the ground truth of the datasets. A natural relationship appears regarding the metrics for the linearity assessment: if the number of dimensions is high, the dataset would likely be classified as non-linear, at least in one of its dimensions. This does not necessarily imply that mildly non-linear methods will fail, but if so, our method would recommend directly applying the binary recursive division method to increase the performance measures of predictive models, despite the higher computational cost.

Our method applies state-of-the-art algorithms in a special order and following a novel strategy to optimize the results, which allows generating classification or regression models in general datasets, especially those addressed in this manuscript as highly-non linear. However, since our method uses previously developed ML methods, we are bound by their own limitations, in the sense that many of the flaws of our method are but a legacy of the ML algorithms used. Taking this into account, we recommend the use of the library and the proposed methodology in datasets with a reduced number of categories in their categorical variables since the library encodes them using One Hot Encoder. The recursive binary partition methodology should not be used when the number of classes is much larger than the available examples, as it may lead to detriments on the performance metrics because of the class balance buffer incorporated in the algorithm.

Future work contemplates the development of a web-based computational tool implementing our methodology, allowing non-specific users to enjoy the advantages of RV-Clustering, without the need to invest time gaining the knowledge that would be required by command-line execution. As the development of predictive models is common to different areas of application, we expect our methodology, library, and the future web-based service, to become a useful tool for the scientific community and a significant contribution to state of the art.

## Data Availability Statement

The https://github.com/dMedinaO/nonlinearModels repository contents the datasets generated and analyzed for this study.

## Author Contributions

DM-O, SC, and ÁO-N: conceptualization. DM-O and ÁO-N: methodology and project administration. DM-O and CQ: validation. DM-O, SC, CQ, and ÁO-N: investigation. DM-O and SC: writing and original draft preparation. SC and ÁO-N: writing, review, and editing. ÁO-N: supervision and funding resources.

### Conflict of Interest

The authors declare that the research was conducted in the absence of any commercial or financial relationships that could be construed as a potential conflict of interest.
